# Tailoring Rational Manufacturing of Extemporaneous Compounding Oral Dosage Formulations with a Low Dose of Minoxidil

**DOI:** 10.3390/pharmaceutics14030658

**Published:** 2022-03-17

**Authors:** Carlos Torrado-Salmeron, Almudena Laguna, Alicia Guillén, Miguel G. Saro, Antonio Matji, Juan J. Torrado, Dolores R. Serrano

**Affiliations:** 1 Department of Pharmaceutics and Food Technology, Complutense University of Madrid, 28240 Madrid, Spain; ctorrado@ucm.es (C.T.-S.); almulagu@ucm.es (A.L.); aligui01@ucm.es (A.G.); misaro@ucm.es (M.G.S.); antonio.matji@cantabrialabs.es (A.M.); 2 Galenical and Industrial Pharmaceutical Institute, Complutense University of Madrid, 28240 Madrid, Spain

**Keywords:** minoxidil, content, uniformity, tablet, capsule, particle size, compounding

## Abstract

Low amounts of minoxidil in oral dosage forms are commonly prescribed as anti-alopecic pharmacological treatments. Side effects are usually related to individual susceptibility. However, poor drug content and mass uniformity can lead to a potential risk of overdosing, and higher chances to experience side effects. The impacts of four formulation variables on drug content and mass pharmaceutical quality attributes were studied with an experimental design at two levels. The first variable (A) was the particle size of the direct compression microcrystalline cellulose (MCC) used as a diluent (Avicel^®^ PH 101 vs. LP 200). The second variable (B) was the type of production process (direct filling vs. wet granulation). The third variable (C) was the particle size of riboflavin added as a color mixture indicator agent (granular vs. milled). The fourth variable (D) was the type of oral solid dosage form (capsule vs. tablet). In half of the formulations, the mean minoxidil content and minoxidil uniformity were out of the specification limits of the Pharmacopoeia, demonstrating the importance of carefully selecting the excipients as well as the utilized process when manufacturing low oral dosage minoxidil formulations. The best minoxidil content uniformity was achieved when using MCC LP 200, wet granulation, granular riboflavin, and capsules. However, tablets are the recommended dosage form when utilizing Avicel^®^ PH 101 or direct filling. Meeting these criteria, the content and mass uniformity are more likely to meet the specification limits of the Pharmacopeia. Techniques such as NIR spectroscopy should be implemented to control the quality of extemporaneous compounding formulations with a low dose of active ingredient.

## 1. Introduction

Minoxidil was first introduced as an antihypertensive treatment in the 1970s. However, the discovery of its common adverse event, hypertrichosis, led to the manufacturing of a topical formulation for promoting hair growth [1]. Although topical minoxidil is an effective treatment for alopecia, there is poor patient compliance due to the need for application twice a day, undesirable hair texture, and scalp irritation [2]. Recently, oral minoxidil, at a low dose between 0.25 and 5 mg, has been proposed as a well-tolerated treatment for hair loss, presenting a lower standard adverse effect rate than standard doses. The most common adverse effects after low oral doses for minoxidil are pedal edema (2%), lightheadedness (1.7%), insomnia (0.2%), postural hypotension (1.1%), and heart rate alterations (1.3%), which force patients to discontinue the treatment [3,4].

Although those side effects are usually related to individual susceptibility, poor content and mass uniformity can lead to a potential risk of overdosing and higher chances to experience side effects. In Spain, most of these prescriptions are formulated as extemporaneous compounding formulations in community pharmacies in small batches of approximately 30 units in the form of capsules of size numbers 3 or 4. Even though the elaboration of this oral formulation must be performed to assure the quality of the finished product, the low dose of minoxidil within the capsules, along with lack of time and poor analytical resources in most community pharmacies, can result in poor drug content uniformity. To overcome this challenge, riboflavin, an orange powder, is commonly incorporated in the powder mixture before capsule filling as a visual color agent indicator to ensure correct mixture homogeneity. However, it has not been demonstrated that just the visual control of the colored riboflavin homogeneity assures an adequate content uniformity for minoxidil, bearing in mind the difference in particle size between both powder substances.

The hypothesis underpinning this work is that the overall manufacturing process of low oral dose minoxidil formulations can have a significant impact on drug content, mass uniformity and, hence, treatment efficacy and toxicity. The selection of the solid dosage form (capsules versus tablets), the production process (direct powder filling versus wet granulation), the particle size of the diluents used, such as microcrystalline cellulose (Avicel^®^ PH 101 vs. LP200 type), and the particle size of the riboflavin added as a color mixture indicator agent (granular vs. milled) may play major roles in the overall minoxidil content uniformity. This work aimed to study the effect of the four different formulation variables mentioned above on the minoxidil content uniformity containing riboflavin as a homogeneity color indicator. The mean minoxidil content and content uniformity may be affected by differences in the particle size of the powders, the mixing conditions, the possibility of wet granulation, and even the selection of the type of oral solid dosage form (capsule or tablet). An experimental factorial design of these four variables at two levels was developed to study the uniformity of minoxidil and riboflavin contents and the mass uniformity with the ultimate goal of tailoring the rational manufacturing of extemporaneous low oral doses of minoxidil formulations. The studied variables were: (A) the particle size of the microcrystalline cellulose employed as a diluent (Avicel^®^ PH 101 vs. LP 200), (B) the elaboration process (direct filling vs. wet granulation), (C) the particle size of the riboflavin added to the powder mixture as a visual indicator of homogeneity (granular vs. milled riboflavin) and (D) the type of dosage form (capsule vs. tablet). A carefully structured design of experiments was implemented to investigate the effect of the four previously described variables as a tool recommended on pharmaceutical development to ensure the quality of drug products [5]. Additionally, chemometric models were developed using NIR spectroscopic data with the aim of determining if this technique could be implemented in compounding to ensure the quality of minoxidil low oral dose formulations.

## 2. Materials and Methods

All chemicals applied in formulations were of European pharmacopeial grade. Two minoxidil suppliers, EP Metapharmaceutical (Barcelona, Spain) and Roig Pharma, (Terrasa, Barcelona, Spain) were used. Two riboflavin suppliers, Fagron (Barcelona, Spain) and Acofarma (Madrid, Spain) were also used. Microcrystalline cellulose was obtained from two different suppliers; Avicel^®^ PH 101 from Fagron (Barcelona, Spain) and Emcocel^®^ LP 200, which was a gift from JRS Pharma (Rosenberg, Germany), were used. Colloidal silicon dioxide (Aerosil^®^ 200) was purchased from Evonik (Darmstadt, Germany). All other chemicals for this study were of analytical grade and were used without further purification.

### 2.1. Experimental Design (DoE) of Oral Minoxidil Formulations

The quality by design approach was used to find the optimal manufacturing conditions of a low-dose minoxidil oral formulation. A 2^4^ simple factorial design was performed using the sign criterion [6] or Minitab^®^ 20.3 (Coventry, UK). The impacts of the four different variables described above were investigated on five responses: the percentage of minoxidil content (compared to the theoretical 100%), mass content, dosage uniformity of minoxidil and riboflavin (%), and mass variation (Table 1). Polynomial regression models were calculated targeting a minimization of the minoxidil content variability when preparing extemporaneous compounding minoxidil oral formulations to reduce undesirable adverse effects [7].

Oral formulations containing minoxidil (0.5 mg), riboflavin (0.5 mg), and a mixture of excipients up to 100 mg were developed according to an experimental design described in Table 1. The mixture of excipients is described by the Spanish National Formulary as excipient number 1 for capsules (Spanish National Formulary, 2020) and contains microcrystalline cellulose (98.05%) and colloidal silicon dioxide (1.95%). Both capsule and tablet formulations had 100 mg theoretical mass content. A mixture of powders was achieved by two processes, either direct powder mixture or wet granulation. The direct mixture was obtained following the process of geometric dilution. The granulation process was performed using a mixture of deionized water: ethanol 96° at 50:50 (*v:v*). Riboflavin was dissolved in the water fraction while minoxidil was dissolved in the ethanol phase. Once the liquid fraction was added slowly to the powder mixture, the wet mass was passed through a 1.6 mm sieve and dried at room temperature in a dark closed room for 24 h. Finally, the dried mass was passed through a 1.0 mm sieve. The direct powder mixture or granules were used to manufacture either size 3 capsules or tablets according to the DoE matrix. Gelatin red capsules of size number 3 were provided by Guinama (Valencia, Spain) and were manually filled by a manual capsule filler machine (Capsunorm, Microcaya, Bilbao, Spain). Tablets were obtained with an eccentric tablet press machine (Korsch EK 0, Berlin, Germany) with two 7 mm circular concave manually operated punches. The means ± standard deviations of tablet height and hardness were 2.68 ± 0.17 mm and 51.09 ± 16.2 N, respectively. Batches for both capsules and tablets were of 20 g of powder mixture, although only 30 units of each dosage form were elaborated.

### 2.2. Particle Size

The sizes of the different raw material particles were measured by laser light diffraction according to the monograph 2.9.31 European Pharmacopoeia (10th ed, 2020). Solid powders (5–10 mg) were added to the sampling cell filled with deionized water at a stirring speed of 30 rpm. A Microtrac 3500 (Microtrac Inc, Montgomeryville, PA, USA) was used to determine the following size parameters (µm): mean number (MN) size, mean volume (MV) size, mean area (MA) size, and the standard deviation (SD) of the volume distribution. The size results of the different assayed particles are reported in Table 2. Details about the different size parameters reported by the analyzer are described in the applications note of P.E. Plantz from Microtrac Inc (http://www.vahitech.com/Assets/MicrotracDataExplinationSheet.pdf, accessed on 22 October 2020).

### 2.3. Minoxidil and Riboflavin HPLC Assay: Determination of Minoxidil Content

A reversed-phase HPLC assay was developed and validated based on the minoxidil assay described in USP (2015). A modular Jasco HPLC equipment with a Jasco PU-1580 pump, a Jasco AS-2050-Plus autosampler fitted to a 100 μL sampling loop, and a UV-visible detector Jasco UV-1575 were used. The wavelength detection was set at 230 nm. The mobile phase was a mixture of methanol:purified water (containing 0.1% sodium 1 heptane sulfonic acid bought from Scharlau ref AC 12420100) at proportions of 50:50 (*v:v*). Finally, 50 µL of ortophosphoric acid 85% was added to adjust 1 L of mobile phase pH to 2.7 ± 0.2. The mobile phase was filtered through 0.45 µm filter (Supor^®^-450, Pall Corporation Ref 60173) and was degassed. The flow rate was fixed at 1 mL/minute. The column was a C18 Zorbax^®^ Eclipse XDB (Agilent) ODS1 4.6 × 150 mm with a 3.5 µm particle size. At these experimental conditions, typical working pressures were around 19.3 MPa, and the retention times for minoxidil and riboflavin were approximately 6.2 and 2.2 min, respectively. The volume of the injection was 10 μL. The calibration ranges for minoxidil and riboflavin in purified water were studied between 0 and 10 µg/mL. The regression coefficients were always higher than 0.99. The typical slopes for minoxidil and riboflavin were 31.9 and 28.7, respectively. Test samples were prepared by adding either a tablet or the content of a capsule in a 100 mL volumetric flask. Then, purified water was added and the mixture was stirred and filtered with a 0.45 µm filter (SFPTFE 0250 45NL) and assayed by HPLC. The minoxidil content (%) was evaluated as the mean of 10 individual dosage units in relation to the theoretical content. This value was considered as response 1 (Table 1 in the DoE).

### 2.4. Uniformity of Dosage Units

The uniformity of the dosage units was calculated based on the European Pharmacopoeia (10th edition, 2020). According to the criteria of Pharmacopoeia, the content uniformity of minoxidil and riboflavin and mass variation were individually tested in 10 units. The coefficient of variation (%) of the mean experimental results is reported in Table 1.

### 2.5. Spectroscopic NIR Data

A microNIR Pro 1700 (Viavi, MBT Brandao, Madrid, Spain) was utilized in the range of 950 to 1650 nm. A blank measurement was carried out before the sample analysis using a 99% 1.25” diffuse reflectance standard. The NIR sample measurements were performed in triplicate by placing the probe directly on the surface of the tablets or the powder previously withdrawn from the capsule. Data acquisition was performed with MicroNIR Pro ES 1700 software (VIAVI Solutions Inc, San Jose, CA, USA). The time required to perform each measurement was less than one minute.

### 2.6. Statistical Data Assay

The results of the experimental design were evaluated according to a sign criterion [6]. Student’s two-tailed paired *t*-test was performed with Excel (Office 365, Microsoft). The DoE was performed using Minitab^®^ 20.3 (Coventry, UK). The multivariate data analysis of the NIR spectroscopic data was performed using Unscrambler^®^ X software (CAMO Software, Oslo, Norway). A pre-processing transformation (data normalization and second derivative Savitzky–Golay with seven points) was used. A support vector machine regression (SVR) and a partial least squares regression (PLSR) were used to correlate the amount of minoxidil determined by HPLC from the samples and the NIR spectra. The Kernel and NIPALS algorithms were used to compute the estimated regression coefficients for the PLSR. The performances of the models were evaluated using the correlation coefficient (R^2^) and the root mean-square error (RMSE) to estimate the fit of the validation and calibration samples [8,9].

## 3. Results and Discussion

### 3.1. Design of Experiments

According to USP, the content of minoxidil tablets should fall within the range of 90–110%, while the content uniformity should be, for most of the solid units, in the range 85–115%, with none outside of the 75–125% range (Eur. Ph. 10th, 2020). Table 1 shows how in our experimental conditions for mean minoxidil content (response 1) and minoxidil content uniformity (response 3) less than half of the formulations complied with the Pharmacopoeia specifications. Although the differences observed for the minoxidil content (response 1) and content uniformity (response 3) should have low clinical relevance, these differences support the practice of medical supervision of patients following these pharmacological treatments. These data illustrate the difficulty of achieving accuracy when preparing minoxidil extemporaneous low-dose oral formulations in clinical practice. Interesting, there is no significant (*p* > 0.1) relationship between the drug (response 1) and mass content (response 2). This lack of a significant correlation between responses 1 and 2 suggests that other factors different from tablet or capsule content weights are related to the differences in drug content. This lack of significance also questions the common practice of simply checking the mass as an indirect control to study drug content uniformity. In the Pharmacopoeias, it is a clear fact that when drug content is low the mass weight is not enough to ensure an adequate drug quality control, and hence, quantitative drug assay is required. However, for all the other responses there were significant (*p* < 0.001) relationships. Higher minoxidil mean content (response 1) was significantly (*p* < 0.001) related with better minoxidil (response 3), riboflavin (response 4) and mass (response 5) uniformities. Mean mass powder (response 2) was significantly (*p* < 0.001) related with poorer minoxidil content uniformity (response 3) but better riboflavin (response 4) and mass (response 5) uniformities. More importantly, the relationships among the different content (responses 3 and 4) and mass (response 5) uniformities were always significant (*p* < 0.001) and directly related. If minoxidil content uniformity (response 3) improved, riboflavin and mass uniformities (responses 4 and 5, respectively) also improved. The good direct correlation between riboflavin (response 4) and minoxidil (response 3) uniformities supports the practice of using a color agent as an indicator of uniformity.

Particle size is a key factor in the uniformity of powder mixtures. Table 2 shows the particle size of the different tested raw materials. The particle size of the two different raw materials suppliers for minoxidil was very similar, at around 50 µm of mean volume size. The supplier Metapharmaceutical was selected for the elaboration of the 16 formulations. In contrast, the riboflavin and microcrystalline cellulose materials were very different depending on the type of material and supplier. For this reason, these different raw materials (MCC and riboflavin) were included as variables 1 and 3 in the experimental design.

The experimental design allowed the study of the comparative relevance of the different variables in the responses and the possible interactions. Table 3 shows the signal criteria of the experiment and the relevance of the different variables and their interactions on the studied responses. According to its effect on the responses, the variables and their possible interactions on the studied responses are ordered from the highest effect:

- Response 1 (minoxidil mean content): B (+96.8) > D (+32.0) > Rest;
- Response 2 (mean mass dosage form): D (+42.7) > AB (−30.3) > C (+19.5) > Rest;
- Response 3 (minoxidil content uniformity): B (−29) > AB (−17.8) > Rest;
- Response 4 (riboflavin content uniformity): A (−70.6) > C (−38.9) > Rest;
- Response 5 (mass uniformity): AD (+6.3) > A (−5.5) > D (−5.3) > Rest.

**Figure 1:**
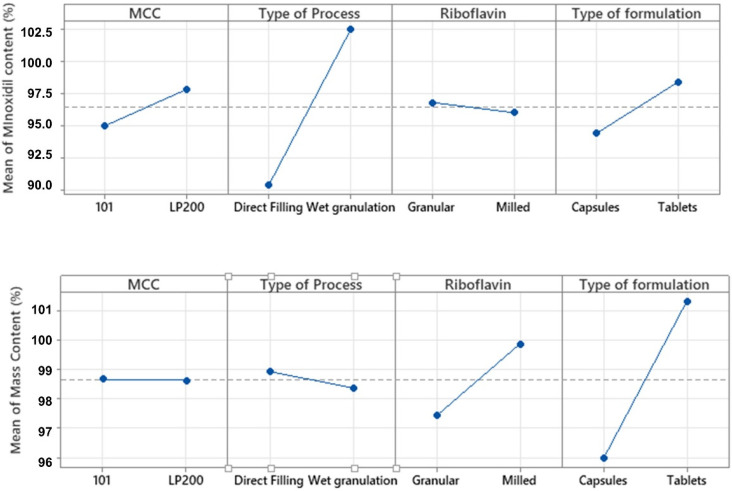
Factorial plots for mean minoxidil content and mass content (%) obtained from Minitab^®^.

The mean and standard deviation values of the different runs for minoxidil content elaborated by direct filling and wet granulation were 0.45 ± 003 and 0.51 ± 0.01 mg/dosage form unit, respectively. The elaboration process (variable B) was the most relevant variable affecting mean minoxidil content. The wet granulation process was significantly (*p* < 0.001) related with a higher mean content of minoxidil. The dosage form (variable D) was also related to a higher mean minoxidil content with mean and standard deviation values of 0.43 ± 0.03 and 0.45 ± 0.03, for capsules and tablets (NS, *p* = 0.11), respectively. The same results were obtained using Minitab^®^ (Figure 1).

Related to the mean mass content (response 2), the most relevant variable was the type of formulation (variable D). The mean contents of tablet weight and mass content of capsule were 96.0 ± 2.3 and 101.3 ± 3.6, for capsule and tablets (*p* = 0.002), respectively. So, in our experimental conditions, capsules exhibited a significantly (*p* < 0.01) lower mean mass than tablets (Figure 1).

Responses 3, 4, and 5 can be compared together because all of them are expressed as coefficient variations in the same units (%). The mean and standard deviations for the minoxidil content (response 3), riboflavin content (response 4), and mass (response 5) uniformities were 8.2 ± 2.8, 15.1 ± 6.5, and 2.1 ± 0.7%, respectively. The worst variations were observed in response 4. The lowest variation was observed for the mass uniformity parameter, while minoxidil and riboflavin content uniformities were approximately four and seven times the mass variation uniformity. Interestingly, there were significant (*p* < 0.001) and direct relationships between these three responses. A higher the mass uniformity (response 5) was significantly related (*p* < 0.001) with both lower minoxidil content (response 3) and lower riboflavin content (response 4) uniformities. These significant and direct relationships among responses 3–5 are indicative of minoxidil and riboflavin uniformity of the mass in the 16 runs.

**Figure 2:**
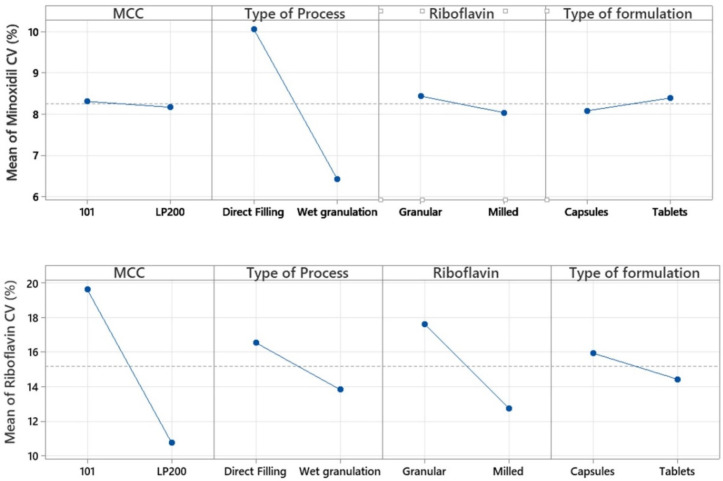
Factorial plots for the mean content uniformity for the minoxidil and riboflavin expressed coefficients of variation (%) obtained from Minitab^®^.

Minoxidil content uniformity (response 3) is a key quality parameter. Table 1 shows how six of the sixteen formulations are outside of the Pharmacopoeia specifications for drug content uniformity. The most efficient way to improve minoxidil content uniformity is by wet granulation (factor B) (Figure 2). The mean and standard deviation values of the different runs for minoxidil content uniformity by direct filling and wet granulation were 10.05 ± 2.49 and 6.43 ± 1.65%, respectively (*p* = 0.005). For the direct filling, the selection of microcrystalline cellulose 101 significantly improves (*p* = 0.08) the minoxidil content uniformity (as an interaction exists between the AB factors in response 3).

Riboflavin content uniformity (response 4) was studied for two reasons: (i) to confirm if riboflavin content is directly related to minoxidil content and (ii) to study the effect of raw material particle size on content uniformity (variable C). Response 4 is directly and significantly (*p* < 0.001) related to minoxidil (response 3) and mass (response 5) uniformities. Therefore, it can be confirmed that the addition of a coloring agent, such as riboflavin, is indicative of the uniformity of the mixture. The importance of particle size on the uniformity of mixture is shown by the crucial effects of diluent particle size (factor A) and riboflavin particle size (factor C) on the riboflavin content uniformity (response 4) (Figure 2). The most efficient way to improve the riboflavin content uniformity was by using the microcrystalline cellulose of a bigger size (factor A). The mean and standard deviation values of the different runs for riboflavin content uniformity with MCC Avicel^®^ PH 101 and MCC LP 200 were 19.61 ± 5.95 and 10.78 ± 3.15%, respectively (*p* < 0.01). The second way to improve the riboflavin content uniformity was by using the riboflavin raw material of a smaller size (milled riboflavin) (factor C). The mean and standard deviation values of the different runs for riboflavin content uniformity with granular and milled riboflavin particles were 17.14 ± 8.03 and 12.76 ± 4.35%, respectively. However, Factor C was not significantly different (*p* = 0.2).

**Figure 3:**
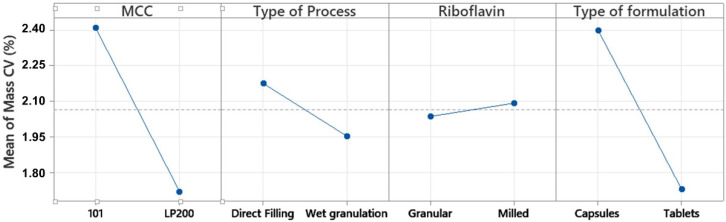
Factorial plots for the mean mass content uniformity expressed coefficients of variation (%) obtained from Minitab^®^.

The most efficient way to improve mass uniformity (response 5) was by the combination of the factors of diluent particle size (factor A) and type of formulation (factor D). Clearly, the worst combination for mass uniformity was capsules prepared with MCC Avicel^®^ PH 101 (interaction AD) (Figure 3). The effect of particle size of diluent was significant (*p* = 0.07). The mean and standard deviation values of the different runs for mass uniformity with MCC Avicel^®^ PH 101 and MCC LP 200 were 2.41 ± 0.89 and 1.72 ± 0.255%, respectively. The type of dosage form (factor D) was also relevant. The mean and standard deviation values of the different runs for mass uniformity with capsules and tablets were 2.40 ± 0.86 and 1.73 ± 0.38%, respectively, and were significantly different (*p* = 0.07).

### 3.2. Formulation Optimization

For optimization purposes, the best minoxidil content uniformity (indicated by the lower coefficient of variation) targeting a 100% drug content was achieved when using MCC LP200, wet granulation, granular riboflavin, and capsules. However, tablets are the recommended dosage form when utilizing MCC Avicel^®^ PH 101 or direct filling. Meeting these criteria, the minoxidil content and coefficients of variation are more likely to meet the specification limits of the Pharmacopeia.

It is worthwhile to note that the use of MCC LP200 is preferable over the MCC Avicel^®^ PH 101 when manufacturing capsules but the opposite is true for the fabrication of tablets. MCC Avicel^®^ PH 101 has a mean particle size close to the raw minoxidil material. The use of excipients of similar particle size is key in achieving a homogenous blend and reducing the potential for de-mixing after the homogenous blend is obtained, especially when a direct filling takes place. However, MCC LP200 demonstrated superior performance in the wet granulation processes. This excipient has excellent flow properties, enabling a homogenous blend to be easily achieved. However, the high potential for the blend to de-mix is not removed, but the wet granulation of this type of blend helps in reducing the de-mixing potential [10,11].

### 3.3. NIR Measurements and Chemometric Models

**Figure 4:**
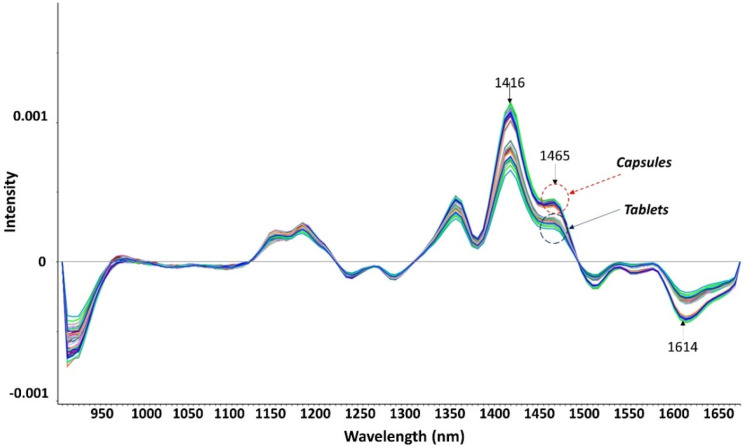
NIR accumulated spectra after data normalization and a second derivative.

In Figure 4, the NIR spectra for the 16 minoxidil batches are illustrated. Differences in three regions, 1416, 1465, and 1614 nm, were detected between capsules and tablets. Chemometric models (SVR and PLSR) were constructed using the signal attributed to these three wavelengths. The content of minoxidil (expressed as the percentage of the theoretical content) was the predicted response.

**Figure 5:**
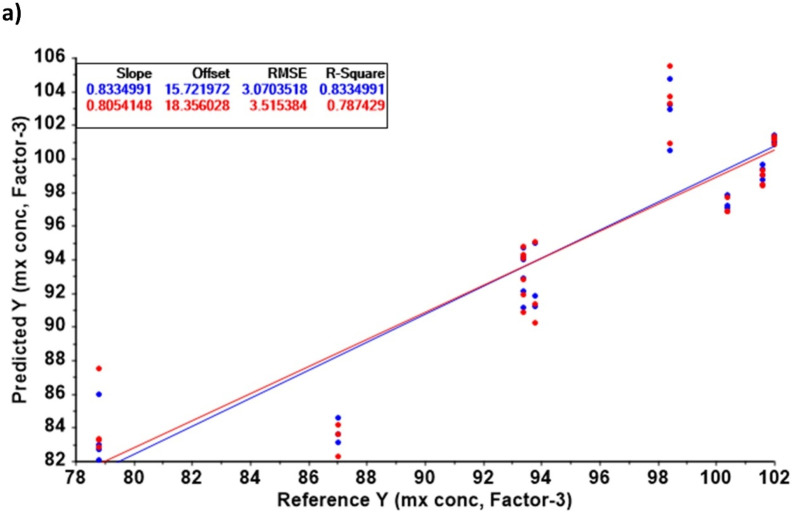

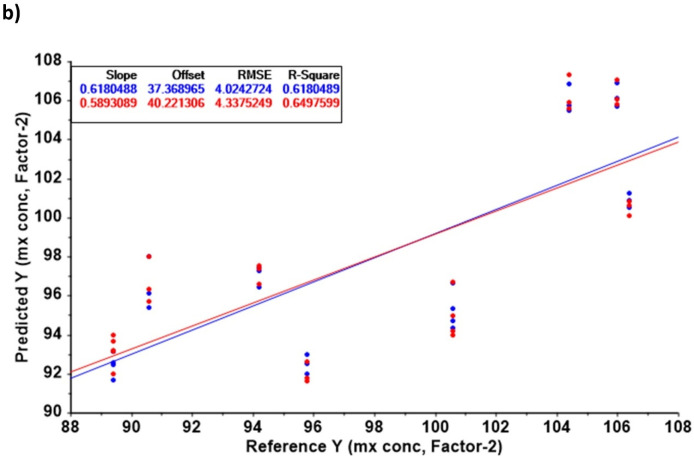
PLSR models for capsules (**a**) and tablets (**b**). Key: mx, minoxidil. Calibration data is represented in blue and validation data in red.

The PLSR showed a better predictive capability of minoxidil content compared to the SVR (Figure 5). To enhance the resolution of the chemometric model, capsules and tablets were plotted separately. A better correlation was observed in the capsules than in the tablets (R^2^ of 0.833 vs. 0.618, respectively). Moreover, the RMSE was 25% smaller for the capsules (3.07 vs. 4.02, respectively). Even though the differences in drug content were small, NIR was demonstrated to have a good sensibility in detecting small differences amongst the 16 prepared batches.

This work highlighted the importance of following a specific protocol to manufacture solid dosage forms with a low amount of drug in order to meet Pharmacopeia specifications. Clinical settings, such as hospitals or community pharmacies, do not possess sufficient analytic techniques to quantify drug content before dispensing the extemporaneous medicines they prepare. Hence, it is essential to ensure that the manufacturing procedure is correct, resulting in a high content uniformity.

In this work, we have analyzed the importance of selecting adequate excipients along with the most suitable manufacturing method to keep an optimal content and mass uniformity of minoxidil low oral dose formulations. Wet granulation showed a superior performance compared to direct mixing and filling of powders when preparing solid dosage forms of minoxidil. However, wet granulation is not a common procedure implemented in local pharmacies, and this is one of the reasons behind the high content variability and, thereafter, side effects. This is the first time that the impact of using wet granulation versus direct filling on the final quality of the solid dosage forms of minoxidil to treat hair loss has been demonstrated. Due to the importance of this issue, it has been addressed before in several patents [12,13]. As an alternative, other authors have investigated the capability of automated powder dispensing systems to fill very low doses of drug directly into capsules (100 µg–5 mg). These systems are designed to dispense pure active pharmaceutical ingredients into the capsules which can be an alternative to the use of wet granulation [14]. However, these systems can be costly, and, hence, a manufacturing technique as described for wet granulation in this manuscript can be an alternative to fabricating extemporaneous low oral dose solid dosage formulations with a high guarantee of meeting the Pharmacopeia specifications. Moreover, the implementation of novel analytical techniques for drug quantification, such as NIR, can be of great use in clinical settings, bearing in mind that they are clean, non-destructive, and 100% of the solid dosage forms can be analyzed in a matter of minutes before dispensing.

## 4. Conclusions

There is a high variability when manufacturing a low oral dose of minoxidil extemporaneous formulation in clinical practice. To minimize this variability, it is recommended to use wet granulation rather than the direct filling of powders. The fabrication of tablets instead of capsules reduces the variability encountered in the mass of the solid dosage forms. The use of MCC LP200 with a larger particle size aids in improving content uniformity. The implementation of NIR tools in compounding could aid in minimizing the risk of high content variability when manufacturing low oral dose solid dosage formulations.

## Figures and Tables

**Table 1:** Experimental conditions of the different tested formulations in the DOE. Responses are: the minoxidil content, mass content, dosage uniformity of minoxidil and riboflavin (%), and mass variation (responses 1–5). Sign criteria and key codes: ^1^ Two different particle sizes of microcrystalline cellulose (MCC) were used fine (Avicel^®^ PH 101) (−) vs. coarse (LP 200) (+); ^2^ Two different mixture processes were tested, direct filling (−) vs. wet granulation (+); ^3^ Two different riboflavin particle sizes were tested: granular (−) vs. milled (+); ^4^ two different dosage forms, capsules (−) vs. tablets (+), were tested; ^5^ Mass content was evaluated as tablet weight or capsule content estimated as the difference between the filled and empty capsules.

	Factor A	Factor B	Factor C	Factor D	Response 1	Response 2	Response 3	Response 4	Response 5
Run	MCC ^1^	Process ^2^	Riboflavin ^3^	Dosage Form ^4^	Content (% Difference of Theorical)	Content (mg/form)	Content Uniformity (CV)	Content Uniformity (CV)	Mass Variation (CV)
					Minoxidil	Mean Mass ^5^	Minoxidil	Riboflavin	Powder
1 *	101	Direct filling	Granular	Capsules	78.8 *	96.0	11.09 *	29.05	3.33
2 *	101	Direct filling	Milled	Capsules	87.0 *	94.7	9.87 *	20.24	3.48
3 *	LP200	Direct filling	Granular	Tablets	94.2	102.2	15.39 *	12.78	1.96
4	101	Wet granulation	Milled	Capsules	100.4	97.4	8.62	14.78	2.55
5 *	LP200	Direct filling	Milled	Tablets	90.6 *	105.1	9.41 *	8.91	1.64
6	101	Wet granulation	Granular	Capsules	101.6	94.7	6.62	24.06	3.19
7	101	Direct filling	Milled	Tablets	89.4	99.8	7.08	15.17	2.4
8	101	Direct filling	Granular	Tablets	95.8	97.7	8.0	23.17	1.8
9 *	101	Wet granulation	Granular	Tablets	106.4 *	101.2	6.45	19.87	1.19
10 *	LP200	Direct filling	Milled	Capsules	93.8 *	98.1	9.36 *	8.25	1.53
11	LP200	Wet granulation	Milled	Tablets	104.4	98.3	6.16	15.71	1.93
12 *	101	Wet granulation	Milled	Tablets	100.6	107.8 *	8.77	10.52	1.34
13 *	LP200	Wet granulation	Granular	Tablets	106.0 *	98.4	5.92	9.45	1.59
14	LP200	Wet granulation	Granular	Capsules	98.4	91.4	3.84	7.86	1.97
15 *	LP200	Direct filling	Granular	Capsules	93.4 *	97.8	10.23 *	14.79	1.27
16	LP200	Wet granulation	Milled	Capsules	102.0	97.7	5.07	8.52	1.88

* Out of Pharmacopoeia specifications: Minoxidil content (90–110% content, minoxidil tablets USP), mean and uniformity mass (±7.5% in tablets and ±10% in capsules Eur. Pharm 10th), minoxidil content uniformity (85–115% or less than 75–125%, Pharm. Eur. 10th).

**Table 2:** Size results of the different tested raw materials, including the mean volume (MV), mean number (MN), mean area (MA), and standard deviation (SD) of mean volume.

Sample	MV (µm)	SD (µm)	MN (µm)	MA (µm)
Minoxidil EP Metapharmaceutical batch 0070320	51.9	41.1	1.45	12.13
Minoxidil Roig Farma batch 0210257	51.75	28.20	0.92	13.11
Riboflavin Fagron batch OF 248098 (granular)	24.17	24.12	0.20	0.65
Riboflavin Acofarma batch 200202 (milled)	0.38	0.22	0.13	0.19
MCC cellulose Avicel^®^ PH 101 Fagron 21C08-H01-00125	54.42	30.85	6.91	32.77
MCC cellulose Emcocel^®^ JRS Pharma LP200 2S6069	445.2	404.15	27.12	101.2
Silicon dioxide Aerosil^®^ 200 154011213	53.69	24.97	19.71	38.47

**Table 3:** Signal criteria of the experimental design and the relevance of the different variables and their interactions on the studied responses. Sign criteria and key codes: Variable A ^1^ Two different particle sizes of microcrystalline cellulose (MCC) were used, fine (Avicel^®^ PH 101) (−) vs. coarse (LP200) (+); Variable B ^2^ Two different mixture processes were tested, direct filling (−) vs. wet granulation (+); Variable C ^3^ Two different riboflavin particle sizes were tested, granular (−) vs. Milled (+); Variable D ^4^ two different dosage forms, capsules (−) vs. tablets (+), were tested. Response 1: mean minoxidil content (% of theorical value), response 2: mean mass content (% of theorical value), responses 3 and 4: minoxidil and riboflavin dosage uniformities (%), and response 5: mass variation uniformity (%).

8	A ^1^	B ^2^	C ^3^	D ^4^	AB	AC	AD	BC	BD	CD	ABC	ABD	BCD	ABCD
1	−	−	−	−	+	+	+	+	+	+	−	−	−	+
2	−	−	+	−	+	−	+	−	+	−	+	−	+	−
3	+	−	−	+	−	−	+	+	−	−	+	−	+	+
4	−	+	+	−	−	−	+	+	−	−	−	+	−	+
5	+	−	+	+	−	+	+	−	−	+	−	−	−	−
6	−	+	−	−	−	+	+	−	−	+	+	+	+	−
7	−	−	+	+	+	−	−	−	−	+	+	+	−	+
8	−	−	−	+	+	+	−	+	−	−	−	+	+	−
9	−	+	−	+	−	+	−	−	+	−	+	−	−	+
10	+	−	+	−	−	+	−	−	+	−	−	+	+	+
11	+	+	+	+	+	+	+	+	+	+	+	+	+	+
12	−	+	+	+	−	−	−	+	+	+	−	−	+	−
13	+	+	−	+	+	−	+	−	+	−	−	+	−	−
14	+	+	−	−	+	−	−	−	−	+	−	−	+	+
15	+	−	−	−	−	−	−	+	+	+	+	+	−	−
16	+	+	+	−	+	+	−	+	−	−	+	−	−	−
**Response 1**	22.80	96.80	−6.40	32.00	−19.20	4.00	−16.80	−3.60	−2.00	−28.40	14.00	14.80	8.80	−11.20
**Response 2**	−0.30	−4.50	19.50	42.70	−30.30	−0.70	−4.70	11.50	6.30	3.50	−5.50	−0.90	−8.50	−9.50
**Response 3**	−1.12	−28.98	−3.20	2.48	−17.82	−7.56	14.28	14.78	3.82	−5.48	1.86	−12.30	4.14	4.10
**Response 4**	−70.59	−21.59	−38.93	−11.97	15.21	31.95	26.83	15.51	12.63	9.01	19.15	−2.61	2.05	3.81
**Response 5**	−5.51	−1.77	0.45	−5.35	3.71	−0.07	6.29	−0.93	−1.73	1.09	1.55	−3.55	1.35	0.67
